# Case Report: Increase in wall shear stress in a narrowed true lumen after type A aortic dissection repair analyzed by computed fluid dynamics

**DOI:** 10.3389/fcvm.2024.1478430

**Published:** 2024-11-07

**Authors:** Yasunori Iida, Yoichi Marushita, Yuka Motohashi, Toshio Sato

**Affiliations:** ^1^ Department of Cardiovascular Surgery, Saiseikai Yokohamashi Tobu Hospital, Yokohama, Japan; ^2^ Department of Clinical Engineering, Faculty of Medical Science and Technology, Gunma Paz University, Takasaki, Japan; ^3^ Department of Clinical Engineering, Faculty of Biomedical Engineering, Toin University of Yokohama, Yokohama, Japan

**Keywords:** computational fluid dynamics, distal stent graft-induced new entry, wall shear stress, frozen elephant trunk, aortic remodelling

## Abstract

A 46-year-old man suffered from acute type A aortic dissection (TAAD) and underwent total arch replacement using the frozen elephant trunk (FET) procedure. During follow-up, we noted back pain and found distal stent graft-induced new entry (dSINE) at the FET distal part by computed tomography. We performed additional extended thoracic endovascular aortic repair (TEVAR) for this pathology. The time between TAAD repair and TEVAR was 2 months. We investigated this complication by computational fluid dynamics analysis through pre- and post-dSINE. The results showed increased wall shear stress at the narrowed true lumen (TL) site, not at the FET site, indicating that prestenotic hydrodynamic load may affect dSINE occurrence.

## Introduction

With the worldwide use of the frozen elephant trunk (FET) procedure, consideration of distal stent graft-induced new entry (dSINE), a major FET complication, has become essential (1). Herein, we investigated wall shear stress (WSS) using computational fluid dynamics (CFD) analysis before and after dSINE occurrence following acute type A aortic dissection (TAAD) repair using the FET procedure. We consider that true lumen (TL) stenosis with poor aortic remodelling contributes to dSINE occurrence.

## Case report

A 46-year-old man with no previous cardiovascular risk factors or medical history suffered from acute type A aortic dissection and underwent total arch replacement using a 26-mm 4-branched prosthetic graft (J Graft: Japan Lifeline, Tokyo, Japan) by the FET procedure using a 27–90 mm Frozenix (Japan Lifeline, Tokyo, Japan). During follow-up, the man suffered from back pain and contrast-enhanced computed tomography (CT) revealed dSINE at the FET. We performed additional extended thoracic endovascular aortic repair (TEVAR) for this pathology. The time between TAAD repair and TEVAR was 2 months. The patient was discharged on postoperative day 4 without any complications.

**Figure 1:**
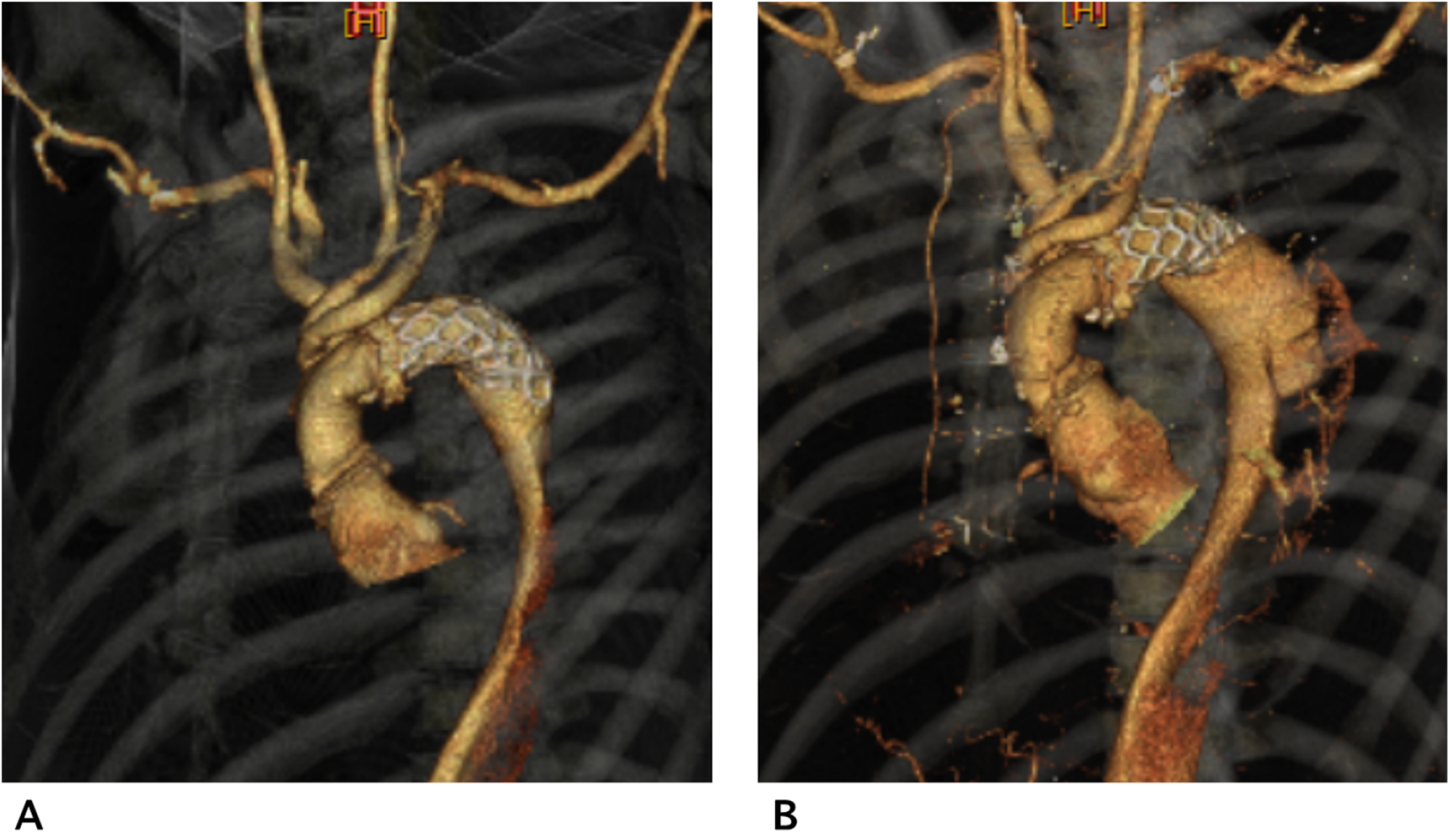
Computed tomography angiography (CTA) after type A aortic dissection repair showed poor remodelling with a narrow true lumen (TL) **(A)** CTA after dSINE, which occurred 2 months later after TAAD repair showed aortic enlargement and a residual narrowed TL **(B)**.

**Figure 2:**
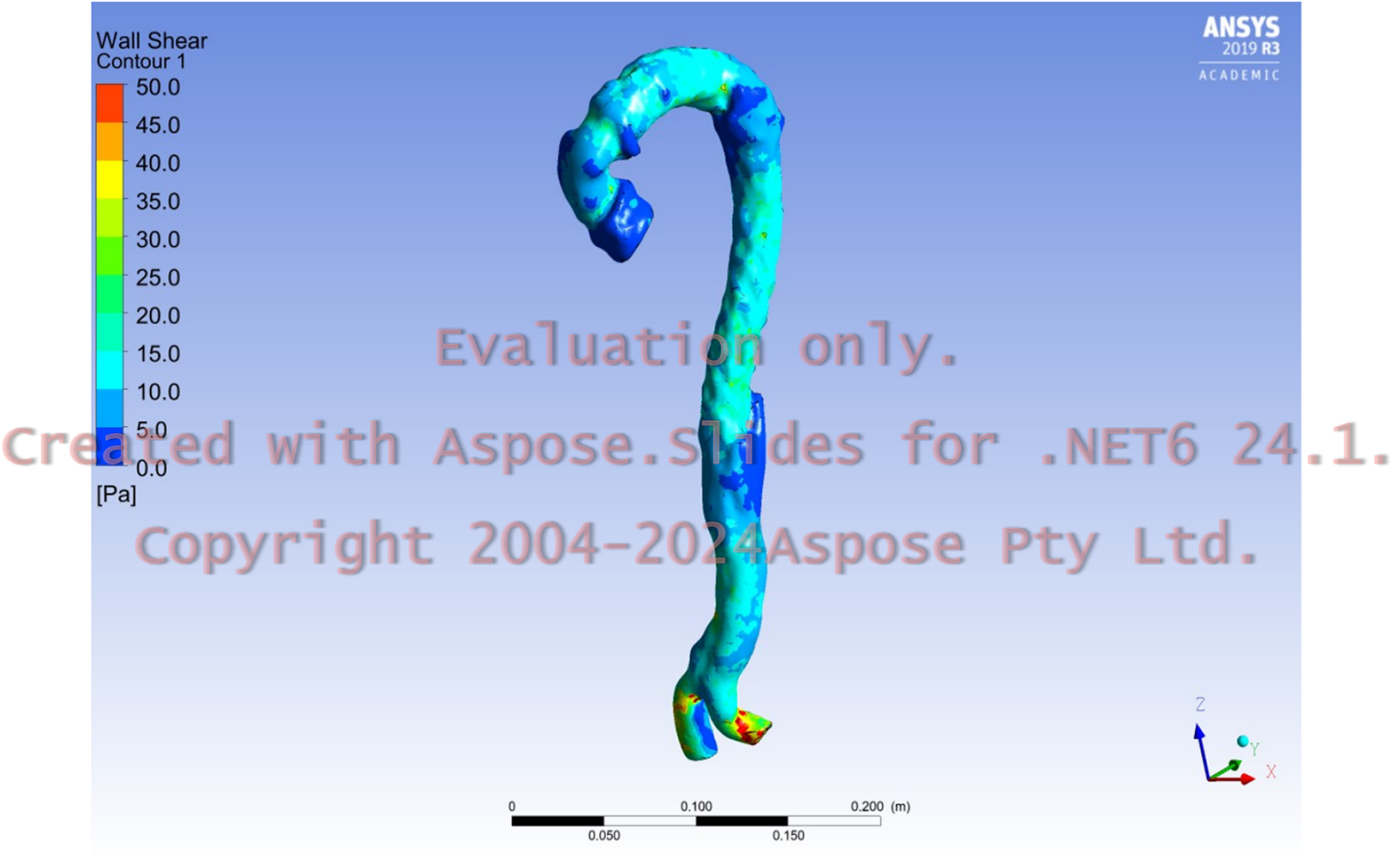
Postoperative wall shear stress after additional extended thoracic endovascular aortic repair showed a consistent decreased value in the descending aorta and downstream aorta, which had been repaired using a stent graft.

We investigated dSINE hydrodynamically using CFD analysis as we had CT data before and after dSINE. Postoperative computed tomography angiography (CTA) after TAAD repair revealed poor aortic remodelling with severe TL stenosis (Figure 1A). CTA after dSINE continued to show poor aortic remodelling and residual TL stenosis (Figure 1B). CFD analysis revealed a marked increase in WSS coinciding with the TL stenosis site of the descending aorta (Supplementary Video 1), which was thought to have caused a pressure gradient before and after this stenotic site. This pressure gradient may have affected the repetitive stimulation between the fragile dissected septum and the FET, causing dSINE (Supplementary Video 2). CFD analysis after additional extended TEVAR showed a WSS decrease at the descending and downstream aorta (Figure 2).

## Discussion

dSINE, a major FET complication, reportedly occurs up to 25% after 3 years (2). When a prosthesis harder than the fragile dissected aorta is inserted, dSINE has been reported even when a graft elephant trunk was used, the so-called (soft-graft)-induced new entry (3).

Herein, we focused on addressing the possible risk factor of postoperative narrowed TL by analyzing the CFD streamline. An increased WSS just before the blood passes through the stenosis site has been shown in blood flow simulations involving patients with coarctation of the aorta (4). Similarly in this case, CFD analysis 7 days after the FET procedure showed a marked increase in WSS in the TL stenosis proximal site. This was an aortic stenosis-like condition, and repetitive heartbeat stimulation and mechanical stress on the solid FET and vulnerable dissected aorta in the distal arch caused dSINE. Osswald et al. retrospectively investigated WSS in dSINE development using CFD. They reported that elevated WSS may lead to aortic wall weakening which may be considered a risk factor for the progression or development of new dissections sites (5). In their review of 5 cases, they found elevated WSS in the descending aorta, suggesting that residual TL stenosis after the FET procedure may affect dSINE development.

## Conclusion

We investigated dSINE hydrodynamically using CFD analysis and considered that the increased WSS due to TL narrowing and the repetitive mechanical contacts between the FET and the dissected and weakened aortic septum could cause dSINE. Poor aortic remodelling with a narrow TL would be a risk factor for dSINE after the FET procedure and TEVAR for type B aortic dissection.

## Data Availability

The original contributions presented in the study are included in the article/Supplementary Material, further inquiries can be directed to the corresponding author.

## Supplementary material

Supplementary Video 1 Computational fluid dynamics (CFD) before dSINE revealed a marked increase in WSS coinciding with the site of true lumen (TL) stenosis of the descending aorta.

Supplementary Video 2 CFD at dSINE onset also showed an increase in WSS at the site of TL stenosis of the descending aorta.
